# A Case–Control Study on the Origin of Atypical Scrapie in Sheep, France 

**DOI:** 10.3201/eid1505.081119

**Published:** 2009-05

**Authors:** Alexandre Fediaevsky, Eric Morignat, Christian Ducrot, Didier Calavas

**Affiliations:** Agence Française de Sécurité Sanitaire des Aliments, Lyon, France (A. Fediaevsky, E. Morignat, D. Calavas); Institut National de la Recherche Agronomique, Clermont-Theix, France (A. Fediaevsky, C, Ducrot)

**Keywords:** Prions and related diseases, scrapie, transmissible spongiform encephalopathy, case–control study, sheep, epidemiology, France, research

## Abstract

A matched case–control study (95 cases and 220 controls) was designed to study risk factors for atypical scrapie in sheep in France. We analyzed contacts with animals from other flocks, lambing and feeding practices, and exposure to toxic substances. Data on the *prnp* genotype were collected for some case and control animals and included in a complementary analysis. Sheep dairy farms had a higher risk for scrapie (odds ratio [OR] 15.1, 95% confidence interval [CI] 3.3–69.7). Lower risk was associated with organic farms (OR 0.15, 95% CI 0.02–1.26), feeding corn silage (OR 0.16, 95% CI 0.05–0.53), and feeding vitamin and mineral supplements (OR 0.6, 95% CI 0.32–1.14). Genetic effects were quantitatively important but only marginally changed estimates of other variables. We did not find any risk factor associated with an infectious origin of scrapie. Atypical scrapie could be a spontaneous disease influenced by genetic and metabolic factors.

## Abstract 2

Risk factors for this disease suggest a noninfectious origin influenced by genetic and metabolic factors.

Atypical scrapie is a transmissible spongiform encephalopathy (TSE) of small ruminants; it was recently defined by the European Food Safety Authority according to phenotypic features (*1*). This disease was identified in 1998 (*2*), and little is known about its etiology and epidemiology (*3*), which contrasts with the etiology and epidemiology of classical scrapie (*4*). Diagnosis of atypical scrapie is impaired by discrepant clinical diagnostic results (*5*) of rapid diagnostic tests and variable accumulation of scrapie prion protein (PrP^Sc^) in the brainstem (*3*). In France, the average apparent prevalence of atypical scrapie detected by active surveillance with tests recommended by the European Food Safety Authority (*6*,*7*) for the detection of this disease in brainstem samples was 6 cases/10,000 tested animals during 2002–2006. This prevalence was comparable elsewhere in Europe (*8*).

The origin of atypical scrapie is still unclear, and whether the disease has an infectious origin remains a major question. This disease has been transmitted experimentally to Tg-mice (*9*) and sheep (*10*), but histopathologic features of atypical scrapie have suggested similarities with human spontaneous TSE (Gerstmann-Sträussler-Scheinker syndrome) (*2*,*11*). The few reports on >1 case of atypical scrapie in the same flock provide insufficient information to draw conclusions on natural transmission of this disease (*3*). If atypical scrapie had an infectious origin, it could be influenced by risk factors associated with a pattern of infectious disease transmission as described for classical scrapie (*12*–*14*). In 2006, a case–control study of atypical scrapie in Norway did not detect such factors, but it showed that the removal of the placenta at lambing could have a protective effect (*15*). Feeding of vitamin and mineral supplements showed an adverse effect, which was interpreted as interaction of some minerals with cellular prion protein (*16*,*17*), rather than a feed contamination. Such an effect warrants confirmation.

Genetic factors should be considered when investigating risk factors for atypical scrapie because some mutations of the *prnp* gene, which codes for prion protein (PrP), modify the risk for this disease (*2*,*18*,*19*). Because all described genotypes of the *prnp* gene confer susceptibility to sheep, a purely genetic origin is unlikely but a confounding effect could occur.

Other possible origins for atypical scrapie could involve exposure to toxic substances, particularly pesticides, which were shown to be involved with other neurodegenerative diseases involving protein disorders such as Parkinson disease and Alzheimer disease (*20*,*21*). Some biochemical mechanisms for these diseases could be similar. To confirm the findings of Hopp et al. (*15*) and to explore further hypotheses on risk factors for atypical scrapie, we conducted a case–control study of sheep in France.

## Materials and Methods

### Study Design

The epidemiologic unit was the animal, and most of the data collected concerned its birth cohort, assuming that in each flock all animals born during the same birth campaign (C_0_, defined from July 1 of year n – 1 to June 30 of year n) shared the same exposure. Cases and controls were matched by frequency matching on their birth cohort (C_0_) so that their distributions were similar over the birth campaigns.

Cases were recruited among cases detected by the active surveillance program during January 2006–March 2007. The index case had to be a female that was born and reared all its life in the same flock, with a known C_0_. A total of 137 cases met these criteria.

Two controls per case were selected. The control animal was an animal born in C_0_, kept until birth campaign 2006 (C_2006_) in the same flock, and originated from a control flock randomly selected among the list of flocks from which >1 sheep had been tested in 2006 with a test recommended for the detection of atypical scrapie. All results from TSE rapid tests on sheep from control flocks had to be negative for atypical scrapie and classical scrapie. Each control had 9 replacement animals randomly selected from sheep flocks from the same county (French département).

Flocks of case and control animals were required to have no history of scrapie and >20 ewes kept for reproduction. Males were not included in the study because they have a low incidence of atypical scrapie and because farming practices used with rams are different from those used with ewes.

### Data Collection

Four persons interviewed farmers during the summer of 2007. The questionnaire, which was available in French on request, was divided in 5 parts: 1) 13 questions on structure and economic context of the flock, 2) 7 questions on purchase of sheep and contacts with other flocks, 3) 3 questions on lambing management, 4) 16 questions on feeding practices including list of feed, and 5) 8 questions on exposure to toxic products, including the list of products used. Questions related to structure of the flock were asked for C_0_ and C_2006_ to check if changes had occurred. Questions related to exposure during the first months of life were asked only for C_0_, questions related to general feed exposure were asked for the period between C_0_ and the 2 subsequent reproduction campaigns (C_0_–C_0+2_) and questions related to exposure to toxic products and mineral feeding were asked for C_0_–C_2006_. For each flock, the number of animals tested with a recommended test for atypical scrapie during active surveillance programs during 2002–2006 was extracted from the Base Nationale des Encéphalopathies Spongiformes Transmissibles Animales.

*Prnp* genotypes at codons 136, 141, 154, and 171 were determined by Labogena (Jouy en Josas; France). For cases, material examined consisted of a sample of soft tissue (muscle or ear) or brainstem. For controls, the matching constraint was relaxed to enable interviewers to sample some hair from any ewe born during C_0_–C_0+2_.

### Data Management

Data were entered into a Microsoft (Redmond, WA, USA) Access 2000 database. All statistical analyses were performed by using R 2.6.1 for Windows (*22*).

Three types of toxic exposure were assessed: pesticides on crops, insecticides on premises, and antiparasitic treatments. For each category, active components of products reported were identified from databases (*23*–*26*). The Direction des Végétaux et de l’Environnement from the Agence Française de Sécurité Sanitaire des Aliments specified the known or suspected neurotoxic components and their mechanism of action. Three categorical variables were created and were assigned a value of 1 for a case of exposure to any neurotoxic product of the category of concern during C_0_–C_2006_ and a value of 0 otherwise. Missing values from the questionnaire were imputed by using available covariates as predictors after verifying that the missingness pattern was compatible with random missing (*27*).

Genotypes were linearly classified into 5 levels of risk on a log scale (Table 1) according to the odds ratio (OR) estimated for the sheep population in France (*19*). Many genotypes were missing, mainly for controls because of difficulties in extracting DNA from hair samples (n = 117) and for a few cases because of unsuitable samples (n = 13). Missing values for controls were randomly imputed by using distribution of genotypes per breed. From the distribution of all available genotypes of cases of atypical scrapie in France, 20 datasets were imputed. To account for geographic distribution of flocks, France was divided into 9 sheep production areas according to sheep farming density and production patterns.

**Table 1:** Genotypes grouped by levels of genetic risk for atypical scrapie in sheep, France*

Group	Genotypes of *prnp* gene	Coded level
1	ALRR-ALRQ, ALRR-VLRQ, ALRQ-ALRQ, ALRQ-ALRH, ALRQ-VLRQ	0
2	ALRR-ALRR, ALRR-ALRH, VLRQ-VLRQ	1
3	ALHQ-ALRH, ALHQ-VLRQ, AFRQ-ALRH, ALRH-ALRH, AFRQ-VLRQ, ALRH-VLRQ	2
4	ALRR-ALHQ, ALRR-AFRQ, ALHQ-ALRQ, AFRQ-ALRQ	3
5	ALHQ-ALHQ, ALHQ-AFRQ, AFRQ-AFRQ	4

*Groups showed homogeneous odds ratios for atypical scrapie. The level of risk is the value of the corresponding log linear variable introduced into the multivariate model. *prnp*, prion protein.

### Univariate and Multivariate Analyses

Analyses were conditional to the matching variable and based on univariate and multivariate generalized linear mixed models with the logit link function for the outcome and C_0_ as a random coefficient (*28*,*29*). ORs and their 95% confidence intervals were derived from the coefficient estimates and variance parameters. When variables could not be introduced simultaneously in the multivariate analysis because they were collinear, the most biologically sound variable was selected.

Variables for the multivariate model were selected according to the recommendations of Hosmer and Lemeshow (*28*). Candidate variables for the multivariate model were backward selected according to the log-likelihood ratio test. Candidate variables with a p value <20% in univariate analyses were tested before other variables were tested. The effect of variables with a p value >20% on the coefficient parameter of the selected variables was then verified 1 at a time. Best parameterization of continuous variables and statistical significance of interactions terms were then checked. A false discovery rate (FDR) (*30*) was calculated by using p values of the log-likelihood ratio tests for tested variables and interaction terms.

A complementary model was used to assess if genetics influenced stability of the final model. For each of the datasets imputed, level of genetic risk was introduced in the final model as an ordinal covariate; coefficients, standard errors, and Wald test p values of different variables were inferred according to the method of Little and Rubin (*27*).

### Sensitivity Analysis

The national database used to sample controls did not enable us to take into account the size of the flocks. Therefore, counties with a large percentage of small flocks (<20 ewes) may have been overrepresented. To assess the influence of geographic selection bias, we conducted a sensitivity analysis by using 2 methods: 1) weighting of controls in the final model with weights being defined for each county as the ratio of the percentage of flocks >20 ewes in the county divided by the percentage of flocks >20 ewes at the national scale, and 2) introduction of sheep production areas as random coefficients in the final model.

## Results

### Study Population

Among 137 selected farms containing cases, 11 did not satisfy the selection criteria. In addition, 11 farmers refused to participate and 20 could not be reached. A total of 95 cases were included in the study. For controls, 1,131 farmers were contacted to participate in the study; 621 controls did not satisfy the selection criteria (374 because flocks had <20 ewes, 41 because matching criteria could not be satisfied, 20 because flocks had <20 ewes and matching criteria were not satisfied, and 186 because of other reasons)**.** In addition, 124 farmers refused to participate and 161 could not be reached. A total of 225 controls were included in the study.

**Figure 1:**
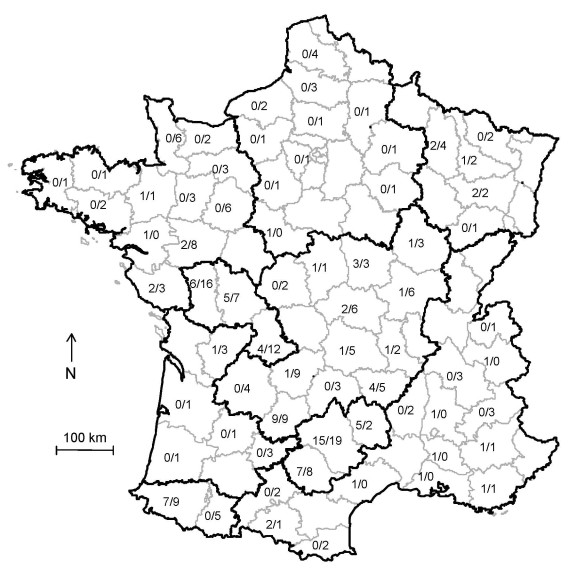
Distribution of cases of atypical scrapie and controls (no. cases/no. controls) in sheep, France, 2007. Sheep production areas are outlined in black, and counties are outlined in gray.

**Figure 2:**
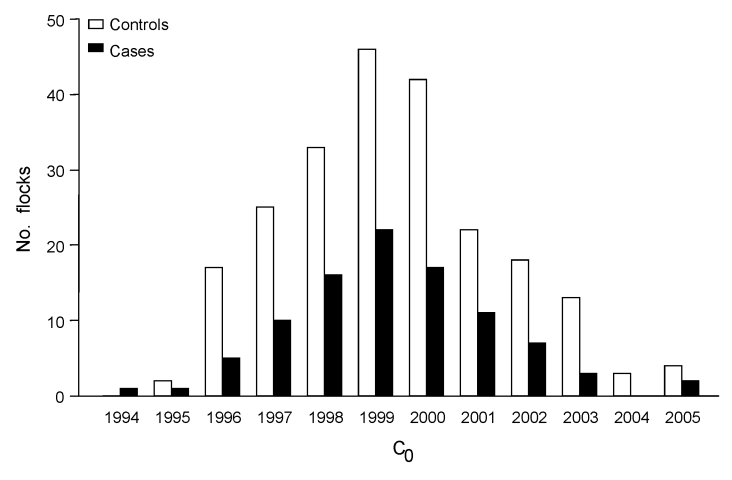
Distribution of C_0_ for cases of atypical scrapie and controls in sheep, France, 1994–2005. C_0_, birth cohort assuming that in each flock all animals born during the same birth campaign (defined from July 1 of year n – 1 to June 30 of year n) shared the same exposure.

The 95 cases and 225 controls were located throughout France (Figure 1). Case animals were born during 1994–2005. Because of exclusion of cases independent of the selection of their matched controls, the average ratio of controls per case was 2.4 instead of 2 and varied according to C_0_ (Figure 2). There were a few missing values for questionnaire variables (0.4%).

### Univariate Analyses

Flocks containing cases were larger than flocks containing controls, had more animals tested for TSEs, and were present more often on sheep dairy farms (Table 2). Moreover, these 3 variables showed a significant correlation (Pearson coefficient of correlation between size of flock and number of animals tested ρ = 0.67, Spearman coefficients of correlation between size of flocks and dairy production ρ = 0.23 and between number of animals tested and dairy production ρ = 0.20, p<0.001). None of the variables associated with a hypothesis of infectious origin was associated with atypical scrapie (Table 3).

**Table 2:** Univariate analyses of farm structure variables conditional to C_0_ for atypical scrapie in sheep, France*

Variable	Controls	Cases	Odds ratio	p value
Mean ± SE no. animals tested during 2002–2006	15 ± 17	32 ± 29	1.04	<0.001
Sheep dairy farm				
No	196	64	3.3	<0.001
Yes	29	31		
Flock of familial origin				
No	70	24		
Yes	155	71	1.3	0.29
Flock of external origin				
No	126	58		
Yes	99	37	0.8	0.41
Member of a producer organization during C_0_–C_2006_				
No	104	35		
Yes	121	60	1.5	0.12
Follow-up of farm results during C_0_–C_2006_				
No	112	36		
Yes	113	59	1.6	0.05
Organic farm during C_0_–C_2006_				
No	211	94		
Yes	14	1	0.2	0.08
Sent flock animals to breeding centers during C_0_–C_2006_				
No	192	63		
Yes	33	32	3.0	<0.001
Presence of cows during C_0_–C_0+2_				
No	115	67	0.4	<0.001
Yes	110	28		
Presence of goats during C_0_–C_0+2_				
No	200	81	1.4	0.36
Yes	25	14		
Presence of pigs during C_0_–C_0+2_				
No	216	93	0.5	0.40
Yes	9	2		
Presence of poultry during C_0_–C_0+2_				
No	209	89	0.9	0.80
Yes	16	6		

*C_0_, birth cohort assuming that in each flock all animals born during the same birth campaign (defined from July 1 of year n – 1 to June 30 of year n) shared the same exposure; SE, standard error; C_2006_, birth campaign 2006; C_0_–C_0+2_, period between C_0_ and the 2 subsequent reproduction campaigns.

**Table 3:** Univariate analyses of contact with sheep from other flocks and afterbirth exposure variables conditional to C_0_ for atypical scrapie in sheep, France*

Variable	Controls	Cases	Odds ratio	p value
Contact with other flocks during C_0_–C_2006_				
No	189	79	1.1	0.85
Yes	36	16		
Purchase of rams during C_0_–C_2006_				
No	30	17	0.7	0.29
Yes	195	78		
Purchase of ewes during C_0_–C_2006_				
No	139	68	0.6	0.10
Yes	86	27		
No. flocks of origin of ewes purchased during C_2005_–C_2006_				
0	139	68	1.0	0.22
1	35	13	0.8	
2	25	9	0.7	
>4	26	5	0.4	
Disposal of placenta in C_0_				
Never	82	37	1.0	0.51
Sometimes	36	19	1.2	
Always	107	39	0.8	
Use of adoption cases in C_0_				
No	41	12	1.5	0.22
Yes	184	83		

*C_0_, birth cohort assuming that in each flock all animals born during the same birth campaign (defined from July 1 of year n – 1 to June 30 of year n) shared the same exposure; C_2006_, birth campaign 2006; C_2005_, birth campaign 2005.

Variables associated with feeding practices were not associated with increased risk for atypical scrapie (Table 4). Feeding milk replacers, which was negatively correlated with dairy production (Spearman coefficient of correlation ρ = –0.41, p<0.001), and feeding corn silage showed an inverse association with atypical scrapie.

**Table 4:** Univariate analyses of feeding component variables during the specified period and conditional to C_0_ for atypical scrapie in sheep, France*

Variable	Controls	Cases	Odds ratio	p value
Lambs fed milk replacers in C_0_				
No	68	42	0.5	0.02
Yes	157	53		
Corn silage in C_0_				
No	195	90	0.4	0.04
Yes	30	5		
Beet root in C_0_				
No	185	86	0.5	0.06
Yes	40	9		
Straw in C_0_				
No	77	23	1.6	0.08
Yes	148	72		
Oil cake in C_0_				
No	164	73	0.8	0.47
Yes	61	22		
Compound feed in C_0_				
No	78	28	1.3	0.37
Yes	147	67		
Grass silage in C_0_				
No	195	77	1.5	0.20
Yes	30	18		
Grain in C_0_				
No	45	18	1.1	0.83
Yes	180	77		
Molasses in C_0_				
No	212	88	1.3	0.59
Yes	13	7		
Vitamin and mineral supplements in C_0_				
No	102	48	0.8	0.40
Yes	123	47		
Salt licks (pure salt) during C_0_–C_2006_				
No	7	2	1.5	0.62
Yes	218	93		
Salt licks with minerals during C_0_–C_2006_				
No	46	28	0.6	0.08
Yes	179	67		
Other ruminants feed during C_0_–C_0+2_				
No	205	90	0.6	0.27
Yes	20	5		
Other ruminants minerals during C_0_–C_0+2_				
No	203	85	1.1	0.84
Yes	22	10		
Pig feed during C_0_–C_0+2_				
No	209	89	0.9	0.80
Yes	16	6		
Poultry feed during C_0_–C_0+2_				
No	193	80	1.1	0.71
Yes	32	15		

*C_0_, birth cohort assuming that in each flock all animals born during the same birth campaign (defined from July 1 of year n – 1 to June 30 of year n) shared the same exposure; C_2006_, birth campaign 2006; C_0_–C_0+2_, period between C_0_ and the 2 subsequent reproduction campaigns.

Pesticides and insecticides on the premises correlated with an increased risk for atypical scrapie (Table 5). These 2 variables correlated with dairy production (Spearman coefficients of correlation ρ = 0.33 and ρ = 0.39, respectively, p<0.001).

**Table 5:** Univariate analyses of exposure to toxic product variables during the specified period and conditional to C_0_ for atypical scrapie in sheep, France*

Variable	Controls	Cases	Odds ratio	p value
Use of mineral drugs during C_0_–C_2006_				
No	99	38	1.2	0.50
Yes	126	57		
Pesticides containing neurotoxic components used on crops during C_0_–C_2006_				
No	155	51	1.9	0.009
Yes	70	44		
Insecticides containing neurotoxic components used on premises during C_0_–C_2006_				
No	169	55	2.2	0.002
Yes	56	40		
Antiparasitic treatments containing neurotoxic components during C_0_–C_2006_				
No	100	47	0.8	0.42
Yes	125	48		

*C_0_, birth cohort assuming that in each flock all animals born during the same birth campaign (defined from July 1 of year n – 1 to June 30 of year n) shared the same exposure; C_2006_, birth campaign 2006.

### Multivariate Analyses

The set of candidate variables included 36 categorical variables and 1 continuous variable (Tables 6, 7). The final model included 5 variables and 1 interaction term (Table 6). The random coefficient had a null variance, and the scale parameter was close to 1, which indicated an absence of shrinkage.

**Table 6:** Multivariate analyses coefficient parameters of the final model for atypical scrapie in sheep, France*

Variable	Coefficient (β)	Standard error (β)	p value†
Random coefficient	0	2.00 × 10^–5^	
Intercept	−1.51	0.24	2 × 10^–10^
No. animals tested during 2002–2006	0.04	0.01	6 × 10^–10^
Sheep dairy farm	2.71	0.78	2 × 10^–5^
Organic farm	−1.88	1.08	0.03
Corn silage in C_0_	−1.81	0.59	5 × 10^–4^
Vitamin and mineral supplements in C_0_	−0.51	0.33	0.02
Interaction term between sheep dairy farm and vitamin and mineral supplements in C_0_	−1.69	0.88	0.04

*For categorical variables, the reference value was no. C_0_, birth cohort assuming that in each flock all animals born during the same birth campaign (defined from July 1 of year n – 1 to June 30 of year n) shared the same exposure. †By log likelihood ratio test.

**Table 7:** Adjusted odds ratios of atypical scrapie associated with variables computed from the final model in sheep, France*

Variable	Adjusted odds ratio	95% CI
No. animals tested increased by 5	1.22	1.11–1.35
Sheep dairy farm when vitamin and mineral supplements not given	15.06	3.25–69.73
Sheep dairy farm when vitamin and mineral supplements given	2.77	1.21–6.37
Organic farm	0.15	0.02–1.26
Corn silage	0.16	0.05–0.53
Vitamin and mineral supplements used on sheep dairy farms	0.18	0.03–1.04
Vitamin and mineral supplements not used on sheep dairy farms	0.6	0.32–1.14

*CI, confidence interval.

No variable associated with a hypothesis of infectious origin was present in the final model. The number of animals tested and sheep dairy farming were associated with disease (Table 7). Organic farms, feeding corn silage, and use of vitamin and mineral supplements showed an inverse association with disease. Use of these supplements, which was not significant by univariate analysis, was significant after adjustment for sheep dairy farming, and the 2 variables had a significant interaction term. The uncontrolled FDR for our analysis was 33%, which indicated that one third of the variables in the final model were spurious.

After we introduced the genetic effect, estimates of other variables did not vary by >25% of their initial values (Table 8). In addition, genetics showed a strong effect; OR for atypical scrapie ranged from 2.6 for genotypes in group 2 to 48.4 for genotypes in group 5 (Table 1).

**Table 8:** Multivariate analysis including genetic risk from multiple imputation parameters for atypical scrapie in sheep, France*

Variable	Coefficient (β)	Standard error (β)	p value†
Intercept	−3.03	0.37	7 × 10^–16^
Level of genetic risk‡	0.97	0.13	1 × 10^–13^
No. animals tested during 2002–2006	0.03	0.01	5 × 10^–5^
Sheep dairy farm	2.52	0.96	8 × 10^–3^
Organic farm	−2.38	1.29	0.07
Corn silage in C_0_	−1.48	0.68	0.03
Vitamin and mineral supplements in C_0_	−0.40	0.40	0.31
Interaction term between sheep dairy farm and vitamin and mineral supplements in C_0_	−1.99	1.09	0.07

*Genetic risk from multiple imputation parameters was estimated by the method of Little and Rubin (*27*). C_0_, birth cohort assuming that in each flock all animals born during the same birth campaign (defined from July 1 of year n – 1 to June 30 of year n) shared the same exposure. †By Wald test. ‡Because the variable is ordinal, the odds ratio (OR) for a given level of genetic risk is the exponential of the coded level (see Table 1) multiplied by the estimated coefficient β. The ORs for groups 2–5 are 2.6, 7.0, 18.4, and 48.4, respectively.

### Sensitivity Analysis

**Figure 3:**
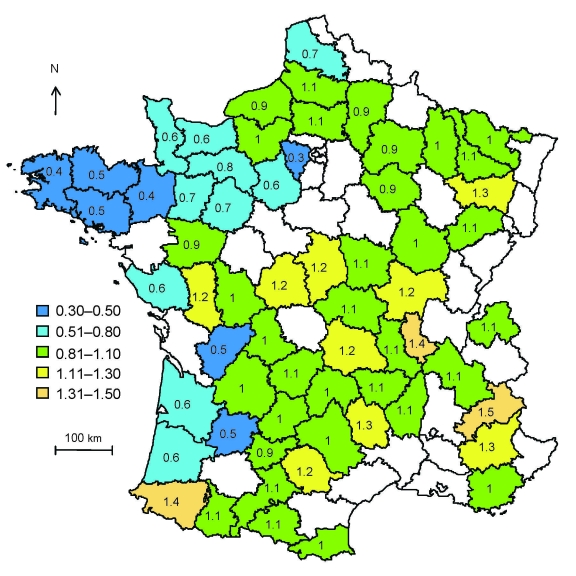
Distribution of control weightings calculated as the ratio of the percentage of flocks with >20 ewes in the county over the average percentage of flocks with >20 ewes for atypical scrapie in sheep, in France, 2007. Ranges represent classes of weightings.

Sensitivity analysis to check possible geographic selection bias led to the same results as analysis without taking into account geographic selection bias when either weighting of samples (Figure 3) or adjusting for sheep production areas (Figure 1) was used. These results indicate that putative bias had no statistical effect on the results.

## Discussion

There was no evidence of a relationship between risk for atypical scrapie and factors related to an infectious origin of the disease in France. Our results are consistent with those of Hopp et al (*15*). The only difference in the study by Hopp et al. was that removal of the placenta was associated with a decreased risk for disease. Other studies of atypical scrapie also suggested that the disease could have a noninfectious origin (*3*). The variables in our dataset were not associated with disease, and their corresponding ORs in univariate and multivariate analyses, regardless of the parameterization of the variables, were ≈1, which suggests that they did not tend to be risk factors.

These results contrasts with those of case–control studies on classical scrapies, which found relationships between risk for disease and introduction of ewes (*12*–*14*), grazing on the same pasture as other flocks (*14*), exposure to placenta (*13*), and feeding concentrates (*12*). Green et al. reported that flocks in which cases of scrapie (atypical or classical) were detected were larger and had more exchange of animals between flocks than control flocks (*31*). When they studied movement of sheep between flocks of equivalent status, flocks positive for classical scrapie were interconnected but flocks positive for atypical scrapie were not. These authors indicated that their results were compatible with atypical scrapie not being infectious.

Our results showed the influence of nutritional and metabolic factors. Although sheep dairy farming covers a broad category of farms with many factors, sheep dairy farms often use more sophisticated technology, and dairy ewes are more exposed to metabolic disorders because of high levels of exported nutrients, including minerals, during milk production. Thus, some feed components such as vitamin and mineral supplements or corn silage could alleviate the risk for disease. This lessening of risk may also occur with less harsh farming conditions found on organic farms.

There is evidence suggesting that minerals, especially copper, manganese, and zinc, could play a role in the physiopathology of prion diseases (*16*,*17*). In contrast with our results, Hopp et al. (*15*) found that feeding of vitamin and mineral supplements was associated with atypical scrapie. The difference in the association between atypical scrapie and vitamin and mineral supplements in the 2 studies could be explained by differences in local conditions or roles of some minerals (*17*). Proper balance of minerals is complex because many interactions occur between mineral intake, diet of the animals, and physiologic conditions. Results of the few epidemiologic studies conducted on this topic (*32*–*35*) were rarely conclusive but such associations are difficult to demonstrate with observational data.

Among different mechanisms that should be explored to understand occurrence of atypical scrapie, we believe that toxic exposure should not be overlooked. In our study, organic farms were less at risk, and univariate analysis showed that pesticides on crops and insecticides on the premises were risk factors for disease. Multivariate analysis showed a confounding effect of dairy farming but this finding should not rule out a possible effect of pesticides. Organophosphates and pyrethroids were frequently identified among reported products; these 2 groups of active components, which may be associated with Parkinson disease (*21*,*36*), interfere with the mitochondrial chain, which is a possible pathway for prion diseases (*37*). Another study on toxic exposures and gene–environment interaction could be more conclusive regarding this issue (*38*). In that regard, flocks recently exposed to insecticide treatment for control of bluetongue vectors could constitute interesting cohorts to follow up for atypical scrapie.

Genetic factors had no confounding effect on variables in the final model, and associated ORs were high (<48 for animals with the highest genetic risk). This high magnitude of risk, compared with other factors, suggests that genetic factors play a role in the epidemiology of atypical scrapie. However, exploring the role of genetic factors in the origin of atypical scrapie would require estimation of penetrance of different mutations, but this estimate is not currently available. In addition, genetic factors are known to be a major risk factor for classical scrapie despite a contagious origin (*4*).

In an exploratory study such as ours, interpretation of variables in the final model is not straightforward. Particular caution should be given to the risk for purely statistical associations and to selection, classification, or confusion biases. The risk for purely statistical associations increases with the number of variables tested. An FDR estimates the rate of such spurious associations (*30*), which was high in our final model (FDR 33%). To limit the FDR to a low value (5%), one would only consider as confident results variables with a p value <6 × 10^–4^, which are the number of animals tested, dairy farming, and use of corn silage. However, a high FDR should not prevent discussion of findings. Rather, this value provides a safeguard against overinterpretation of results when testing many hypotheses.

We identified a geographic selection bias in selection of controls. However, sensitivity analysis indicated that this bias did not influence the results. Misclassification problems for some variables could not be excluded, especially those regarding recall bias and memory failure. To minimize these problems, farming documents and account books were used when available, and some data were directly obtained from national databases.

Year of birth could have been a confounding factor because cases of atypical scrapie are usually found in old animals, and exposure to risk factors could be time-dependant. We matched controls on the birth cohort and accounted for year of birth as a random coefficient in a generalized linear mixed model that is recommended with this design (*28*,*29*). Null variance of the random coefficient indicated that there was no variation of risk between different birth cohorts.

In several studies on scrapie (atypical or classical), size of the flock was a risk factor for disease (*12*–*14*,*31*,*39*,*40*). There are many possible relationships between size and status of the animals. In the hypothesis of infectious origin, larger flocks are more exposed because of increased contacts, whereas in the hypothesis of spontaneous disease, expectancy of the number of cases increases with the size of the flock. Farming practices could also affect risk for developing disease and could be linked to the size of the flock. Moreover, larger flocks have higher number of animals tested as part of active surveillance, which increases the probability of detecting an animal with disease. The number of animals tested is determined by a combination of many structural factors that involve the size of the flock, local conditions of implementation of the surveillance program, mortality and culling rates, and use of a TSE qualification program. We could not simultaneously adjust for size of the flock and number of animal tested, and we prioritized control of surveillance bias. However, the 2 options were numerically equivalent.

Genetic analysis suggested no confounding effect but a strong association with the disease. However, results must be interpreted with caution because sensitivity analysis was conducted after imputing missing data for 53% of the controls and 13% of the cases.

Our final model suggested that atypical scrapie in sheep could be a spontaneous disease with a genetic determinant and possible influence of environmental and metabolic factors. On the basis of our results, there was no risk factor linked to an infectious origin. In particular, atypical scrapie is unlikely to originate from purchase of sheep. Other epidemiologic approaches such as spatial analyses or surveys on occurrence of secondary cases could help substantiate these findings. If infectious origin is confirmed, this finding would indicate that movement limitations of animals from flocks positive for atypical scrapie would not be a key measure in controlling the disease.

## References

[1] European Food Safety Authority. Opinion of the scientific panel on biological hazards on classification of atypical transmissible spongiform encephalopathy (TSE) cases in small ruminants (question no. EFSA-Q-2005–073) adopted on 26 October 2005. Report of the Working Group. The European Food Safety Authority Journal. 2005;276:1–30.

[2] Benestad SL, Sarradin P, Thu B, Schönheit J, Tranulis MA, Bratberg B. Cases of scrapie with unusual features in Norway and designation of a new type Nor98. Vet Rec. 2003;153:202–8. 12956297 10.1136/vr.153.7.202

[3] Benestad SL, Arsac JN, Goldmann W, Noremark M. Atypical/Nor98 scrapie: properties of the agent, genetics, and epidemiology. Vet Res. 2008;39:19. 18187032 10.1051/vetres:2007056

[4] Detwiler LA, Baylis M. The epidemiology of scrapie. Revue Scientifique et Technique de l’Office International des Epizooties. 2003;22:121–43. 10.20506/rst.22.1.1386 12793776

[5] Konold T, Davis A, Bone G, Bracegirdle J, Everitt S, Chaplin M, Clinical findings in two cases of atypical scrapie in sheep: a case report. BMC Vet Res. 2007;3:2. 10.1186/1746-6148-3-2 17298670 PMC1810526

[6] European Food Safety Authority. Scientific report of the European Food Safety Authority on the evaluation of rapid post mortem TSE tests intended for small ruminants. Parma (Italy): The Authority; 2005. Section 31. p. 1–17.

[7] European Food Safety Authority. Scientific report of the European Food Safety Authority on the evaluation of rapid post mortem TSE tests intended for small ruminants (2). Parma (Italy): The Authority; 2005. Section 49. p. 1–46.

[8] Fediaevsky A, Tongue SC, Noremark M, Calavas D, Ru G, Hopp P. A descriptive study of the prevalence of atypical and classical scrapie in sheep in 20 European countries. BMC Vet Res. 2008;4:19. 10.1186/1746-6148-4-19 18544152 PMC2442063

[9] Le Dur A, Beringue V, Andréoletti O, Reine F, Lai TL, Baron T, A newly identified type of scrapie agent can naturally infect sheep with resistant PrP genotypes. Proc Natl Acad Sci U S A. 2005;102:16031–6. 10.1073/pnas.0502296102 16239348 PMC1276041

[10] Simmons MM, Konold T, Simmons HA, Spencer YI, Lockey R, Spiropoulos J, Experimental transmission of atypical scrapie to sheep. BMC Vet Res. 2007;3:20. 10.1186/1746-6148-3-20 17725818 PMC2025597

[11] Buschmann A, Biacabe A-G, Ziegler U, Bencsik A, Madec J-Y, Erhardt G, Atypical scrapie cases in Germany and France are identified by discrepant reaction patterns in BSE rapid tests. J Virol Methods. 2004;117:27–36. 10.1016/j.jviromet.2003.11.017 15019257

[12] Philippe S, Ducrot C, Roy P, Remontet L, Jarrige N, Calavas D. Sheep feed and scrapie, France. Emerg Infect Dis. 2005;11:1274–9. 16102318 10.3201/eid1108.041223 PMC3320489

[13] Healy AM, Hannon D, Morgan KL, Weavers E, Collins JD, Doherty ML. A paired case-control study of risk factors for scrapie in Irish sheep flocks. Prev Vet Med. 2004;64:73–83. 10.1016/j.prevetmed.2004.06.002 15325763

[14] Hopp P, Ulvund MJ, Jarp J. A case-control study on scrapie in Norwegian sheep flocks. Prev Vet Med. 2001;51:183–98. 10.1016/S0167-5877(01)00225-2 11535279

[15] Hopp P, Omer MK, Heier BT. A case-control study of scrapie Nor98 in Norwegian sheep flocks. J Gen Virol. 2006;87:3729–36. 10.1099/vir.0.81951-0 17098991

[16] Choi CJ, Kanthasamy A, Anantharam V, Kanthasamy AG. Interaction of metals with prion protein: possible role of divalent cations in the pathogenesis of prion diseases. Neurotoxicology. 2006;27:777–87. 10.1016/j.neuro.2006.06.004 16860868

[17] Leach SP, Salman MD, Hamar D. Trace elements and prion diseases: a review of the interactions of copper, manganese and zinc with the prion protein. Anim Health Res Rev. 2006;7:97–105. 10.1017/S1466252307001181 17389057

[18] Moum T, Olsaker I, Hopp P, Moldal T, Valheim M, Benestad SL. Polymorphisms at codons 141 and 154 in the ovine prion protein gene are associated with scrapie Nor98 cases. J Gen Virol. 2005;86:231–5. 10.1099/vir.0.80437-0 15604451

[19] Moreno CR, Moazami-Goudarzi K, Laurent P, Cazeau G, Andréoletti O, Chadi S, Which PrP haplotypes in a French sheep population are the most susceptible to atypical scrapie? Arch Virol. 2007;152:1229–32. 10.1007/s00705-007-0956-7 17426916

[20] Baldi I, Lebailly P, Mohammed-Brahim B, Letenneur L, Dartigues JF, Brochard P. Neurodegenerative diseases and exposure to pesticides in the elderly. Am J Epidemiol. 2003;157:409–14. 10.1093/aje/kwf216 12615605

[21] Brown TP, Rumsby PC, Capleton AC, Rushton L, Levy LS. Pesticides and Parkinson’s disease—is there a link? Environ Health Perspect. 2006;114:156–64. 16451848 10.1289/ehp.8095 PMC1367825

[22] R Development Core Team. R: a language and environment for statistical computing 2008 [cited 2009 Feb 12]. Available from http://www.biosino.org/R/R-doc/Rm/README.R-2.6.1

[23] Ministère de l’Agriculture et de la Pêche (MAP). E-phy. 2008 [cited 2008 Jul 17]. Available from http://e-phy.agriculture.gouv.fr

[24] Agence Française de Sécurité Sanitaire des Aliments. Direction du végétal et de l’environnement. Agritox. 2008 [cited 2008 Jul 17]. Available from http://www.dive.afssa.fr/agritox/index.php

[25] Meissonnier E, Devisme P, Join-Lambert P. Dictionnaire des médicaments vétérinaires et des produits de santé animale, 14th ed. Maison-Alfort (France): Editions du Point Vétérinaire; 2007.

[26] Footprint. The FOOTPRINT pesticide properties dataBase. Database collated by the University of Hertfordshire as part of the EU-funded FOOTPRINT project (FP6-SSP-022704) 2007, 2008 [cited 2008 Jul 17]. Available from http://www.eu-footprint.org/ppdb.html

[27] Little RJ, Rubin DB. Statistical analysis with missing data, 2nd ed. New York: John Wiley and Sons, Ltd; 1988.

[28] Brown H, Prescott R. Applied mixed models in medicine. London: John Wiley and Sons, Ltd; 2006.

[29] Agresti A. Categorical data analysis, 2nd ed. New York: Wiley-Interscience; 2002.

[30] Benjamini Y, Yekutieli D. The control of the false discovery rate in multiple testing under dependency. Annals of Statistics. 2001;29:1165–88. 10.1214/aos/1013699998

[31] Green DM, Del Rio Vilas VJ, Birch CP, Johnson J, Kiss IZ, McCarthy ND, Demographic risk factors for classical and atypical scrapie in Great Britain. J Gen Virol. 2007;88:3486–92. 10.1099/vir.0.83225-0 18024920 PMC2884981

[32] Gudmundsdottir KB, Sigurdarson S, Kristinsson J, Eiriksson T, Johannesson T. Iron and iron/manganese ratio in forage from Icelandic sheep farms: relation to scrapie. Acta Vet Scand. 2006;48:16. 10.1186/1751-0147-48-16 16987395 PMC1569367

[33] Imrie CE, Korre A, Munoz-Melendez G. Spatial correlation between the prevalence of transmissible spongiform diseases and British soil geochemistry. Environ Geochem Health. 2009;31:133–45. 10.1007/s10653-008-9172-y 18427934

[34] Chihota CM, Gravenor MB, Baylis M. Investigation of trace elements in soil as risk factors in the epidemiology of scrapie. Vet Rec. 2004;154:809–13. 15260441 10.1136/vr.154.26.809

[35] Slivarichova D, Mitrova E, Koscova S, Uhnakova I. Slovak accumulation of genetic Creutzfeldt-Jakob disease with exogenous risk factor. In: Neuroprion 2007 Conference; 2007 Sep 26–28, Edinburgh, United Kingdom. Fontenay-aux-Roses (France): Commissariat à l’Energie Atomique. p. 3.57–3.61.

[36] Kamel F, Hoppin JA. Association of pesticide exposure with neurologic dysfunction and disease. Environ Health Perspect. 2004;112:950–8. 15198914 10.1289/ehp.7135 PMC1247187

[37] Choi SI, Ju W-K, Choi E-K, Kim J, Lea H-Z, Carp RI, Mitochondrial dysfunction induced by oxidative stress in the brains of hamsters infected with the 263 K scrapie agent. Acta Neuropathol. 1998;96:279–86. 10.1007/s004010050895 9754961

[38] Coppede F, Mancuso M, Siciliano G, Migliore L, Murri L. Genes and the environment in neurodegeneration. Biosci Rep. 2006;26:341–67. 10.1007/s10540-006-9028-6 17029001

[39] Gubbins S, Clark AM, Eglin RD, Sivam SK. Results of a postal survey of scrapie in the Shetland Islands in 2003. Vet Rec. 2006;158:255–60. 16501156 10.1136/vr.158.8.255

[40] McIntyre KM, Gubbins S, Sivam SK, Baylis M. Flock-level risk factors for scrapie in Great Britain: analysis of a 2002 anonymous postal survey. BMC Vet Res. 2006;2:25. 10.1186/1746-6148-2-25 16887027 PMC1557843

